# Construction of an Immune Escape-Related Signature in Clear Cell Renal Cell Carcinoma and Identification of the Relationship between IFNAR1 and Immune Infiltration by Multiple Immunohistochemistry

**DOI:** 10.3390/cancers15010169

**Published:** 2022-12-28

**Authors:** Kun Chang, Fujiang Xu, Xuanzhi Zhang, Bohan Zeng, Wei Zhang, Guohai Shi, Dingwei Ye

**Affiliations:** 1 Department of Urology, Fudan University Shanghai Cancer Center, Shanghai 200032, China; 2 Department of Oncology, Shanghai Medical College, Fudan University, Shanghai 200032, China; 3 Department of Oncology, The Affiliated Hospital of Southwest Medical University, Luzhou 646000, China

**Keywords:** clear cell renal cell carcinoma, IFNAR1, immune escape, biomarker, immunotherapy

## Abstract

**Simple Summary:**

This research found that a higher immune escape score was significantly correlated with a shorter survival time. Meanwhile, through the validation of the external cohort and the correlation analysis of the immune microenvironment, we proved that IFNAR1 is the key gene regulating immune escape in ccRCC, and we also found that the function of IFNAR1 in promot-ing immune activation is achieved by facilitating the infiltration of CD4^+^ T cells and CD8^+^ T cells. Our research found a new key molecule that regulates the immune mi-croenvironment of ccRCC, which could accurately predict the efficacy of ccRCC im-munotherapy, thus providing a new idea for immunotherapy in ccRCC.

**Abstract:**

Background: In the past decade, immunotherapy has been widely used in the treatment of various tumors, such as PD-1/PD-L1 inhibitors. Although clear cell renal cell carcinoma (ccRCC) has been shown to be sensitive to immunotherapy, it is effective only in several cases, which brings great obstacles to anti-tumor therapy for patients. Lawson et al. have successfully identified 182 “core cancer innate immune escape genes” whose deletion makes cancer cells more sensitive or resistant to T-cell attack. Methods: In this research, we sought to explore genes closely associated with ccRCC among the 182 core cancer innate immune escape genes. We used online databases to screen mutated genes in ccRCC, and then used ConsensusClusterPlus to cluster clinical samples to analyze differences in clinical prognosis and immune components between the two subgroups. In addition, the immune escape score was calculated using lasso cox regression, and a stable tumor immune escape-related nomogram was established to predict the overall survival of patients. Results: Higher immune escape score was significantly correlated with shorter survival time. Meanwhile, through the validation of the external cohort and the correlation analysis of the immune microenvironment, we proved that IFNAR1 is the key gene regulating immune escape in ccRCC, and we also found that the function of IFNAR1 in promoting immune activation is achieved by facilitating the infiltration of CD4^+^ T cells and CD8**^+^** T cells. IFNAR1 regulates the malignant behavior of ccRCC by inhibiting the proliferation and migration properties. Conclusions: IFNAR1 may become a key biomarker for evaluating the efficacy of ccRCC immunotherapy and may also be a potential target for immunotherapy.

## 1. Introduction

The occurrence and progression of tumors is a complex process, in which the immune system plays a pivotal role in monitoring and eliminating tumor cells [1]. In the past ten years, immunotherapy has been widely used in the treatment of various tumors, such as PD-1/PD-L1 inhibitors, and its efficacy has been affirmed by researchers. However, not all cancers are sensitive to immunotherapy, and drug resistance often occurs, which brings great obstacles to anti-tumor therapy for patients. Meanwhile, Lawson et al. [2] have screened six different mouse tumor cells using CRISPR technology and successfully identified 182 “core cancer innate immune escape genes” whose deletion makes cancer cells more sensitive or more resistant to T cell attack resistance. 

Renal cell carcinoma (renal cell carcinoma, RCC) is a kind of malignant tumor that accounts for more than 90% of renal cell carcinoma. The prognosis of renal cell carcinoma is poor: 30% of patients have lymph node or distant metastasis at the time of diagnosis, and about 30% of patients will find metastasis during the follow-up [3,4]. RCC could be classified into three main histological subtypes: clear cell RCC (ccRCC), papillary RCC (pRCC), and chromophobe cell RCC, of which ccRCC is the most common subtype (70–85%) [4,5,6], and accounting for the majority of RCC deaths [7]. RCC is very insensitive to chemotherapy and radiotherapy, and surgery is still the first choice for the treatment of RCC. Currently, the main operations for localized renal cell carcinoma are radical nephrectomy (RN) and nephron-sparing surgery (NSS) [8]. For patients with advanced metastatic clear cell renal cell carcinoma (cc-mRCC), a combination of targeted antiangiogenic drugs (such as sorafenib, sunitinib, and other tyrosine kinase inhibitors) and immune checkpoint blockade (such as PD -1/PD-L1/CTLA4 inhibitor) is generally used. Although ccRCC has been proven to be sensitive to immunotherapy, it is only effective in some cases [8]. We speculate that the difference in the efficacy of immunotherapy in different ccRCC patients may be related to the immune escape gene proposed by Lawson et al.

Interferon (IFN) is a pleiotropic cytokine that plays an important role in the development and treatment of cancer [9]. Type I interferons (IFN-1), such as interferon-α and interferon-β, as key mediators in the immune response, have been proven to play a direct role in controlling cell growth, and have potent immunomodulatory and antiangiogenic properties. IFNAR1 (Interferon Alpha And Beta Receptor Subunit 1) is one of the subunits that constitute the type I interferon receptor. It has been reported that proline deletion in IFNAR1 could impair type I interferon signaling, thereby modulating the antitumor immune function of type I interferons [10]. Due to the important function of IFNAR1 in the transmission of interferon signaling, we speculate that IFNAR1 may also play an important role in immune regulation.

In this study, we attempted to explore genes closely related to ccRCC among the 182 core cancer innate immune escape genes screened by Lawson et al. We used online databases to screen mutated genes in ccRCC and then used ConsensusClusterPlus to cluster clinical samples to analyze differences in clinical prognosis and immune components between the two subgroups. In addition, the immune escape score was calculated using lasso cox regression, and a stable tumor immune escape-related nomogram was established to predict the overall survival of patients. Finally, we found IFNAR1 as a key molecule associated with immune escape in ccRCC by external cohort validation. High expression of IFNAR1 was not only significantly correlated with longer survival of patients, but also indicated a high infiltration of various immune cells, especially CD4^+^ T cells and CD8^+^ T cells. The above studies indicated that IFNAR1 may become a key biomarker for the efficacy of ccRCC immunotherapy and may serve as a potential target for immunotherapy.

## 2. Materials and Methods

### 2.1. Data Collection and Normalization

In this study, we enrolled 745 ccRCC samples with transcriptional data and corresponding clinical information after filtering (Repeated samples, samples with incomplete expression, and samples with a follow-up time of fewer than 30 days were deleted). Ninety samples were obtained from Clinical Proteomic Tumor Analysis Consortium (CPTAC) cohort (https://cptac-data-portal.georgetown.edu/study-summary/S050 (accessed on 6 March 2022)). Fifty-two samples were obtained from EMBL’s ArrayExpress database (https://www.ebi.ac.uk/arrayexpress/ (accessed on 15 March 2022)). Ninety samples were obtained from International Cancer Genome Consortium (ICGC) database (https://dcc.icgc.org/ (accessed on 15 March 2022)). Additionally, 513 samples were obtained from the cancer genome atlas (TCGA) cohort (https://portal.gdc.cancer.gov/ (accessed on 25 March 2022)). We merged these samples into one expression matrix and the batch effect was corrected with ComBat from the R package sva [11].

### 2.2. Exploring the Genomic Alterations of Core Cancer Immune Escape Genes

As reported before, Lawson et al. [2] successfully identified 182 “core cancer innate immune escape genes” whose deletion makes cancer cells more sensitive or more resistant to T cell attack resistance (Listed in Table S1). In this study, we first explored the genomic alterations of the core cancer immune escape genes based on the somatic mutation data from the TCGA cohort. Gene Set Cancer Analysis (GSCA, http://bioinfo.life.hust.edu.cn/GSCA/#/ (accessed on 18 April 2022)) is an integrated platform for genomic, pharmacogenomic, and immunogenomic gene set cancer analysis [12]. We explored the somatic alterations of the 182 core cancer innate immune escape genes and a waterfall plot was used to show the top 10 genes with the highest mutation rate. The top 10 genes with the highest heterozygous and homozygous copy number variation were also displayed.

### 2.3. Identification of the Immune Escape-Related Subgroups in ccRCC

Based on the gene expression of the core cancer innate immune escape genes, we utilized ConsensusClusterPlus [13] package to identify potential subgroups. A principle component analysis (PCA) plot was used to show the sample differences. Survival curves were drawn to compare the overall survival of the subgroups. Gene set variation was utilized to find the biological changes between the immune escape-related (IER) clusters. The expression matrix of all genes was extracted and combined with the reference gene set c2.cp.kegg.v7.4 symbol as the input file of the GSVA package [14] in R, and the enrichment score of each gene set in each sample was obtained. Then, the gene set difference analysis was carried out by using the LIMMA software package [15] in R, and the screening threshold was set as the Benjamini–Hochberg correction *p* < 0.05. The first 20 differential gene sets were extracted, and the concentrated fraction heat map of the differential gene set was drawn using the ComplexHeatmap package [16] in R, with grouping and data source tags. Single Sample GSEA (ssGSEA) was performed to explore the associations between tumor microenvironment (TME) and the IER cluster.

### 2.4. Further Optimization of the IER Cluster

We calculated the fold change of the immune escape genes between the two IER clusters and functional enrichment analysis (reference: Gene Ontology and Kyoto Encyclopedia of Genes and Genomes) was utilized to explore the potential biological function of the most changed immune escape genes by using clusterProfiler package [17]. Genes with logFC ≥ 1 and *p*-value < 0.05 was considered significant differentially expressed genes (DEG). Then, we further identify the subgroups (gene Cluster A and gene Cluster B) based on the expression of the DEGs using ConsensusClusterPlus package. Survival curves were also drawn to compare the overall survival of gene clusters. 

### 2.5. Calculating the Immune Escape Score and Construction of Immune-Related Nomogram

Lasso Cox regression was utilized to further optimize the prognostic model and we construct the formula for calculating immune escape score (IE score): IE score = −0.223 ∗ IFNAR1 expression −0.286 ∗ RGP1 expression. We also compared the IE score between different IER clusters and gene clusters. Single Sample GSEA (ssGSEA) was also performed to explore the associations between tumor microenvironment and the IE score. Univariate and multivariate regression analyses were also used to assess the prognostic significance of IE score, grade, clinical stage, T stage, and M stage. We also constructed a nomogram to enhance the clinical usage of the IE score, and receiver operating curves (ROC) were also drawn to evaluate the stability of the biomarkers. 

### 2.6. Exploring the Potential Ability for Predicting Immunotherapy Response of the Biomarkers and TME Associations

There are 180 patients treated with Nivolumab in CheckMate 025 trial and the corresponding gene expression data was obtained from the online supplemental data (online supplemental Table S4) appended to the published paper [18]. We calculated the IE score, and the Kaplan–Meier method was used to explore the prognostic significance of IE score, RGP1 expression, and IFNAR1 expression. The optimal cut-off value was set by using R software. We further explore the associations between IFNAR1 expression and immune checkpoint molecules including CTLA4, LAG3, and PDCD1 by searching the TISIDB [19] database. TISIDB is a web portal for tumor and immune system interaction, which integrates multiple heterogeneous data types. We also evaluate the associations between IFNAR1 expression and tumor-infiltrating immune cells including B cells, CD8^+^ T cells, and CD4^+^ T cells by using the TIMER [20] tool (TIMER is a comprehensive resource for systematical analysis of immune infiltrates across diverse cancer types).

### 2.7. Using Multiplex Immunohistochemistry to Explore IFNAR1 Expression and TME

We obtained tissue microarrays from Shanghai Wellbio Biotechnology Co., Ltd. (Wellbio Biotechnology Co., Shanghai, China). To explore the associations between tumor microenvironment (especially CD4^+^ and CD8^+^ T cells) and IFNAR1 expression level, multiplex immunohistochemistry (mIHC) was performed with anti-human CD4 (Abcam, ab133616), CD8 (Abcam, ab217344), and IFNAR1 (ABclonal, A18594) antibodies. Additionally, we used Visiopharm software (Visiopharm A/S, Hersholm, Denmark) to quantify the expression level of IFNAR1, CD4 and CD8.

### 2.8. Cell Culture and Transfection

Two human ccRCC cell lines (786O and 769P) were preserved in our laboratory. They were cultured in RPMI-1640 medium (HyClone, Logan, UT, USA), supplemented with 10% fetal bovine serum (FBS, Gibco, Carlsbad, CA, USA), and 1% penicillin-streptomycin solution. Cells were cultured with 5% CO_2_ at 37 °C. To find the function of IFNAR1 in ccRCC, 786O, and 769P cells were transfected with either negative shRNA (sh-Con) or shRNA-1/shRNA-2 specifically targeted knockdown *IFNAR1* or were transfected with either empty overexpression vector or IFNAR1-overexpression plasmids (IFNAR1-OE), using Lipofectamine 3000 reagent (Invitrogen) according to the manufacturer’s instructions.

### 2.9. CCK-8 Assays

A total of 2 × 10^3^ 786O or 769P cells were seeded into each well of 96-well plates. The proliferation of 786O and 769P cells from day 1 to day 5 (every day) were detected by CCK-8 (Dojindo, Kumamoto, Japan) in accordance with the manufacturer’s experiment procedures. In brief, 10 μL CCK8 solution was added to each well, and the cells were cultured for 1 h. The OD value of each well was measured at 450 nm using a spectrophotometer. All experiments were conducted in triplicate.

### 2.10. Transwell Assays

A total of 2 × 10^4^ cells suspended in 200 µL FBS-free medium were placed in the upper well of Boyden Transwell chambers (8 μm, 24-well format; Corning Co., New York, NY, USA) which were inserted into a 24-well plate, and culture medium supplemented with 10% FBS was added to the bottom chamber. Cells were cultured for 48 h at 37 °C, then the cells on the surface of the upper well were wiped off and cells on the lower surface were fixed with 4% paraformaldehyde solution and stained with 0.1% crystal violet. 

## 3. Results

### 3.1. Copy Number Variation of Core Cancer Innate Immune Escape Genes in ccRCC

**Figure 1:**
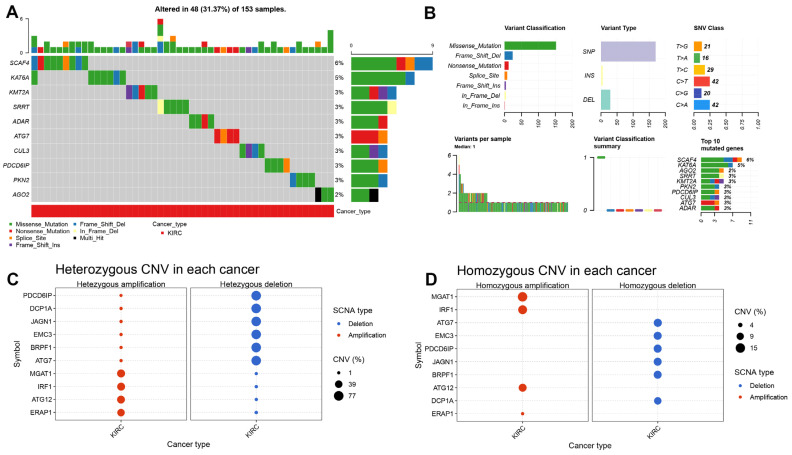
Copy number variation of core cancer innate immune escape genes in ccRCC. (**A**) The top 10 genes with the highest mutation rate in ccRCC. (**B**) Genomic alteration of immune escape genes in ccRCC. (**C**) The top 10 genes with the most significant heterozygous CNVs in ccRCC. (**D**) The top 10 genes with the most significant homozygous CNVs in ccRCC.

**Figure 2:**
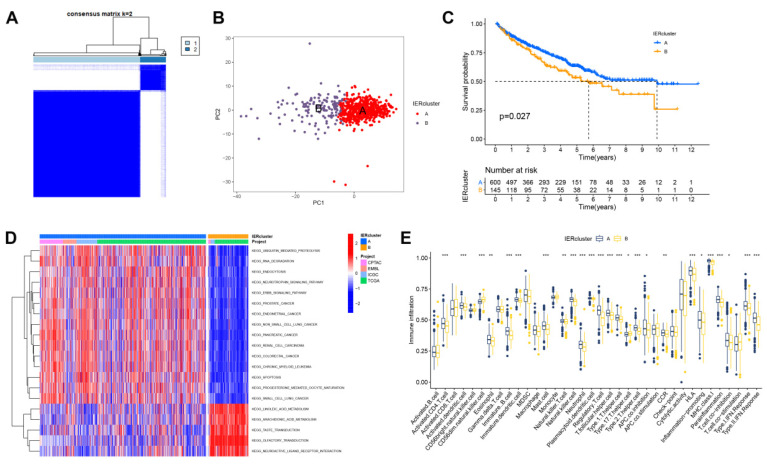
Identification of immune escape clusters. (**A**,**B**) Consensus clustering of ccRCC samples based on immune escape genes. (**C**) Overall survival curves of IER A and IER B clusters using the Kaplan–Meier method. (**D**) Heatmap of biological differences between IER A and IER B clusters. (**E**) Differences in tumor immune microenvironment composition between IER A and IER B clusters (*: *p* < 0.05; **: *p* < 0.01; ***: *p* < 0.001).

In order to screen the altered immune escape genes, we firstly used the online database Gene set cancer analysis (GSCA) to explore the genes with copy number variation (CNV) in ccRCC. As shown in Figure 1A, different forms of mutation in the above immune escape genes existed in 133 ccRCC samples in the TCGA database. The top 10 genes with the highest mutation rate had been listed in Figure 1A, various forms of mutations in these genes occurred in 48 of 133 ccRCC samples, accounting for 31.37% of the total. Missense mutations account for the majority of all forms of mutation in ccRCC, and most mutations occur in single nucleotide polymorphism (SNP). The point mutation in ccRCC usually changes from base C to base T and base C to base A, accounting for about 50% of all mutations (Figure 2B). We next analyzed the copy number amplification or deletion of 182 immune escape genes in ccRCC. Figure 1C,D show the top 10 genes with the most significant heterozygous or homozygous CNVs, respectively.

### 3.2. Identification of Immune Escape Clusters

We extracted the expression of 150 immune escape-related genes (IERs) from 745 ccRCC samples from 4 cohorts of CPTAC (90 samples), EMBL (52 samples), TCGA (90 samples), and ICGC (513 samples). We used the ConsensusClusterPlus method to find potential clusters by assessing the expression levels of IERs (Figure 2A). The ConsensusClusterPlus results indicated that the ccRCC samples could be defined into two clusters, including the IER A cluster and the IER B cluster (Figure 2B). The overall survival of the IER B cluster was significantly shorter than that of IER A cluster (*p* = 0.027, Figure 2C). We also compared the biological differences between the two IER clusters; IER A cluster was significantly enriched in ubiquitin-mediated protein degradation, RNA degradation, and endocytosis, whereas it was significantly decreased in linoleic acid and arachidonic acid metabolism (Figure 2D). Since immune cells and immune-related molecules play important roles in the tumor microenvironment, we assessed TME differences between different clusters with the ssGSEA method. As shown in Figure 2E, the IER A cluster had much higher infiltration of activated CD4^+^ T cells and activated dendritic cells. The IER A cluster also showed higher activity in type I and type II IFN responses. Therefore, TME differences between IER clusters are complex and require to be further study.

### 3.3. Exploring Potential Biological Function of Immune Escape-Related Genes in ccRCC

**Figure 3:**
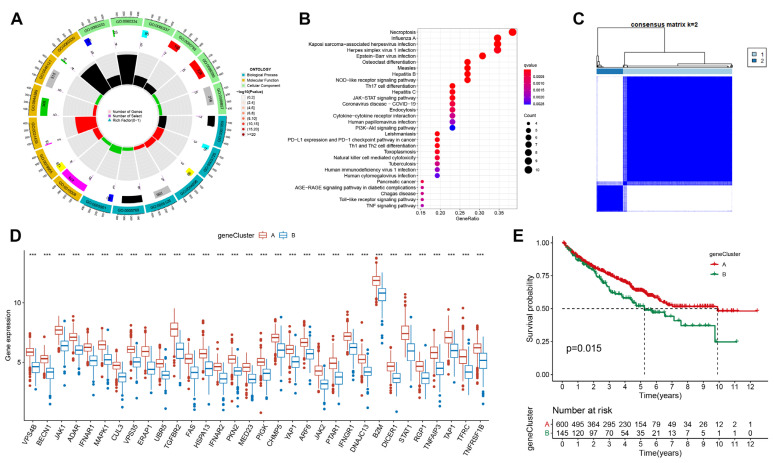
Explore the potential biological function of immune escape-related genes. (**A**) GO enrichment of immune escape-related differential genes (DEGs) between IER A and IER B clusters. (**B**) KEGG enrichment of differential genes (DEGs) between IER A and IER B clusters. (**C**) Quadratic consensus clustering of ccRCC sample usage based on DEGs. (**D**) Expression difference of DEGs between geneCluster A and geneCluster B (***: *p* < 0.001). (**E**) Overall survival curves of geneCluster A and geneCluster B using the Kaplan–Meier method.

To majorize the prognostic model, we further identified differentially expressed genes (DEGs) by comparing expression levels between IER clusters. GO functional enrichment analysis showed that DEGs were mainly enriched in GO:0060333, GO:0060330 and GO:0031435 (Figure 3A). We also explored the potential biological functions of immune escape-related genes; KEGG enrichment found that DEGs were significantly associated with necroptosis, influenza A, Kaposi sarcoma-associated herpesvirus infection, and herpes simplex virus 1 infection (Figure 3B). Based on the expression of genes with the most significant changes, we identified two immune escape gene-related sample clusters (geneCluster). ConsensusClusterPlus defined two clusters of ccRCC based on the expression levels of DEGs between immune escape clusters (Figure 3C). Figure 3D depicts the differential expression of the above differential genes in two geneClusters: geneCluster A and geneCluster B. We further analyzed the association between the two geneClusters and clinical parameters. The overall survival of geneCluster B patients was significantly shorter than geneCluster A (Figure 3E). Therefore, geneCluster B may be a subgroup with more aggressive tumors.

### 3.4. Calculate the Immune Escape Score and Explore Potential Clinical Implications

**Figure 4:**
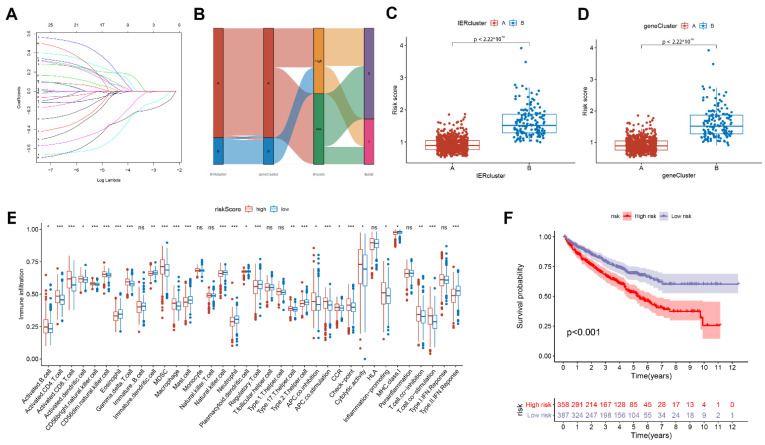
Calculate the immune escape score and explore potential clinical implications. (**A**) Calculation of immune escape scores (IE scores) for ccRCC samples using lasso cox regression. (**B**) Sankey diagram of the relationship between IE scores and IER A and IER B clusters, geneCluster A and geneCluster B, and survival status of ccRCC samples. (**C**) Scatter plot of the relationship between IE scores and IER A or IER B clusters. (**D**) Scatter plot of the relationship between IE scores and geneCluster A or geneCluster B. (**E**) Differences in tumor immune microenvironment composition between high IE score group and low IE score group. (**F**) Overall survival curves of high IE score group and low IE score groups using the Kaplan–Meier method. (*: *p* < 0.05; **: *p* < 0.01; ***: *p* < 0.001).

To improve the clinical translational value of the immune escape-related prognostic model, we calculated immune escape scores using lasso cox regression (Figure 4A). We divided patients into low and high immune escape score groups according to the median of the calculated immune escape scores (IE scores), and analyzed the associations between IER clusters, geneCluster clusters, and IE scores. As shown in Figure 4B–D, the Sankey diagram and scatter plot illustrate that most of the patients in the IER A cluster and geneCluster A belonged to the low IE score group, and the patients in the IER B cluster and geneCluster B belonged to the high IE score group. Next, we analyzed the differences in TME between the high and low IE score groups. As shown in Figure 4E, the low IE score group contained more activated CD4^+^ T cells, activated CD8^+^ T cells, and activated dendritic cells. In addition, we also evaluated the correlation between IE scores and clinical prognosis, as shown in Figure 4F, the overall survival of the high IE score group was significantly shorter than that of the low IE score group.

### 3.5. Construction of Related Immune Escape Nomogram

**Figure 5:**
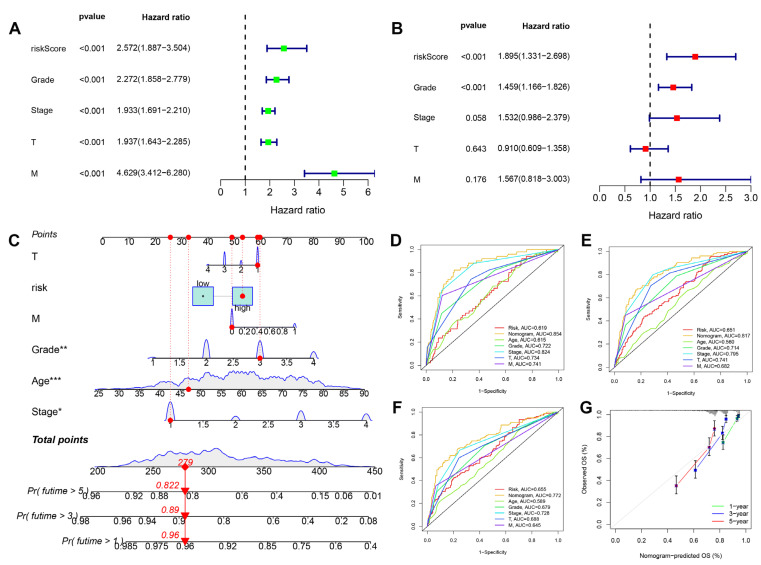
Construction of related immune escape nomogram. (**A**,**B**) Univariate and multivariate cox regression analysis of the prognostic significance of IE scores, grade, stage, T stage and M stage of tumors. (**C**) Construction of a nomogram related to immune escape (*: *p* < 0.05; **: *p* < 0.01; ***: *p* < 0.001). (**D**–**F**) ROC curve of IE score, nomogram, age, grade, stage, T and M in 1 year, 3 years, and 5 years. (**G**) Calibration Chart for testing the accuracy of nomogram in predicting the overall Survival time of ccRCC patients.

In order to evaluate the prognostic significance of immune escape score, we also discussed the stability of the predictive ability of immune escape score. Univariate (Figure 5A, *p* < 0.001, HR = 2.572) and multivariate (Figure 5B, *p* < 0.001, HR = 1.895) cox regression analysis showed that immune escape score could be used as an independent risk factor. We further constructed a nomogram related to the immune escape score (Figure 5C) and tested the stability of the nomogram. The AUC of the nomogram in 1 year, 3 years, and 5 years were 0.854, 0.817, and 0.772, respectively (Figure 5D–F). As shown in the calibration chart (Figure 5G), the OS predicted by the nomogram was very close to the observed OS, so the nomogram can accurately predict the overall survival of the patient.

### 3.6. The Expression Level of IFNAR1 Is Closely Related to Immunotherapy Response and Immune Microenvironment

**Figure 6:**
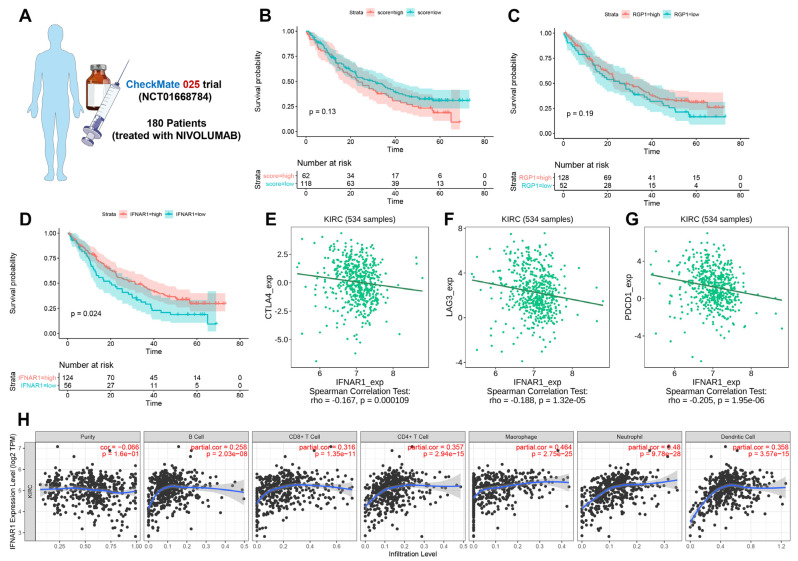
The correlation between the expression level of IFNAR1 with immunotherapy response and immune microenvironment. (**A**) Summary of the patients in external validation. (**B**) Overall survival curves of high IE score group and low IE score groups using the Kaplan–Meier method. (**C**) Overall survival curves of patients with high and low expression of RGP1 using the Kaplan–Meier method. (**D**) Overall survival curves of patients with high and low expression of IFNAR1 using the Kaplan–Meier method. (**E**–**G**) The relationship between the expression of IFNAR1 and the immune checkpoints CTLA-4, LAG-3, and PDCD1, respectively. (**H**) Correlation between IFNAR1 and various types of immune cell infiltration.

To explore whether an immune escape-associated profile correlates with immunotherapy response, we selected 180 ccRCC patients enrolled in the CHECKMate 025 trial (NCT01668784) who received the immune checkpoint PD-1 inhibitor nivolumab for external validation of our established nomogram (Figure 6A). After we performed lasso cox regression analysis, we found that the IE score could be calculated only according to the expression levels of RGP1 and IFNAR1. Therefore, we analyzed the relationship between IE score, RGP1 expression and IFNAR1 expression levels, and the prognosis of the above patients, respectively. As shown in Figure 6B–D, there was no significant difference in overall survival between the two groups of patients with high and low IE scores and between patients with high and low expression of RGP1. However, the overall survival rate of patients with high expression of IFNAR1 was significantly longer than that of patients with low expression of IFNAR1 patients, which proves that IFNAR1 may be closely related to the immunotherapy of ccRCC patients. We further explored the correlation between IFNAR1 expression and immune checkpoint expression in 534 ccRCC samples and found that IFNAR1 expression was negatively correlated with CTLA-4, LAG-3, and PDCD1, respectively (Figure 6E–G). In addition, we also found that the expression of IFNAR1 was significantly positively correlated with the infiltration of CD4^+^ T cells, CD8^+^ T cells, macrophages, neutrophils, and dendritic cells, demonstrating that the high expression of IFNAR1 was closely related to the TME of ccRCC (Figure 6H).

### 3.7. IFNAR1 Promotes Immune Activation by Facilitating CD4^+^ T Cell and CD8^+^ T Cell Infiltration

**Figure 7:**
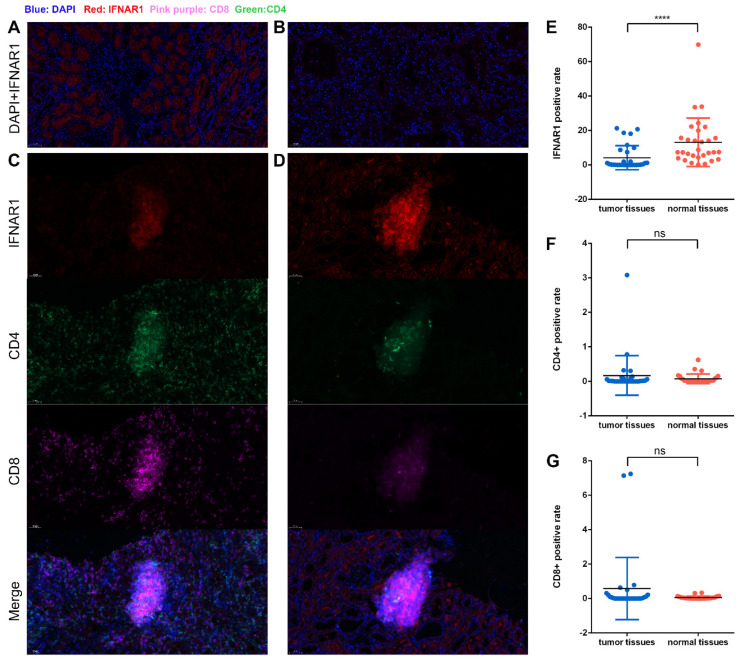
The expression of IFNAR1, CD4 and CD8 molecules in tissues detected by multiplex immunohistochemistry. (**A**) The expression level of IFNAR1 in para-tumor tissues. (**B**) The expression level of IFNAR1 in ccRCC tissues. (**C**,**D**) The association of IFNAR1 expression level with CD4 and CD8 molecules. (**E**–**G**) The quantification of IFNAR1, CD4, and CD8 signals (****: *p* < 0.0001).

To further confirm the anti-tumor immune escape function of IFNAR1, we performed multiplex immunohistochemistry on 30 pairs of ccRCC and para-tumor tissues to observe the expression of IFNAR1 in different types of cells in the tissues (Quantification results were listed in Table S2). We found that the expression of IFNAR1 in ccRCC tissues (Figure 7B) was significantly lower than that in para-tumor tissues (Figure 7A). Meanwhile, as depicted in Figure 7C,D, IFNAR1 was co-expressed with both CD4 and CD8 molecules, indicating that the expression of IFNAR1 is closely related to the infiltration of CD4^+^ T and CD8^+^ T cells, proving that IFNAR1 is a key mediator in promoting immune activation in ccRCC. We also summarized the quantification of the Immunofluorescence signals in Figure 7E–G, and the quantification results also indicated a significantly decreased expression of IFNAR1 in ccRCC tissues. While it is hard to find differences between the CD4/CD8^+^ T cells infiltration in tumor or para-tumor tissues. 

### 3.8. IFNAR1 Regulates the Proliferation and Migration Properties of ccRCC 

**Figure 8:**
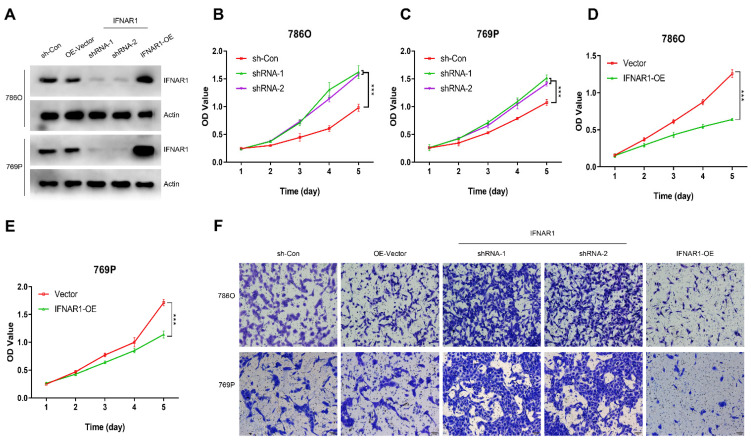
IFNAR1 inhibits the proliferation and migration capacities of ccRCC cells. (**A**) Western blotting assay of IFNAR1 expression in transfected 786O and 769P cell lines. (**B**–**E**) CCK-8 assays showed the effect of IFNAR1 knockdown (**B**,**C**) and overexpression (**D**,**E**) on the proliferation of 786O (**B**,**D**) and 769P (**C**,**E**) cells. (n = 6, error bars are mean ± SD, *** *p* < 0.001 using two-tailed Student’s *t*-test). (**F**) Representative images of the migration assays performed using 786O and 769P cells. Scale bars, 100 µm.

Subsequently, we explored whether IFNAR1 could regulate the malignant behavior of ccRCC. Firstly, we measured the expression level of IFNAR1 in 786O and 769P cells after being transfected with empty vector, shRNA-1, shRNA-2, or overexpression plasmids. It was demonstrated that the expression level of IFNAR1 was decreased in the shRNA-1-transfected group and shRNA-2-transfected group, and significantly increased in the IFNAR1-overexpression-transfected group, in both 786O and 769P cells (Figure 8A). To evaluate the potential function of IFNAR1, we assessed cell proliferation by using a CCK-8 assay. Down-regulation of IFNAR1 promoted cell proliferation and overexpression of IFNAR1 inhibited cell proliferation compared to the control group cells (Figure 8B–E). By conducting the transwell assays, we found that depletion of IFNAR1 remarkably accelerated the migration abilities of 786O and 769P cell lines, whereas IFNAR1 overexpression decreased the migration property (Figure 8F). 

## 4. Discussion

Tumor immunity has become a research hotspot in the field of oncology in recent years [21]. The immune system plays a dual role in cancer: it not only kills cancer cells or inhibits tumor growth, but also promotes tumor progression by creating conditions in the tumor microenvironment that are conducive to tumor growth and metastasis [22,23]. Cancer immunosurveillance refers to the process by which various types of immune cells and mediators recognize and eliminate malignant cancer cells during tumor progression. However, cancer cells can alter the host immune system to evade immunosurveillance, grow and metastases gradually, and then manifest clinical symptoms, which is a vital strategy for tumor survival and development [24,25,26,27]. It is now generally believed that the mechanism of tumor immune escape is the persistent chronic immune stimulation caused by tumor antigens, exhausting T cells and losing most of their effector properties, and the exhausted T cells upregulate PD-1, CTLA-4, LAG-3, and other immune checkpoints [28,29,30]. In addition, other immune cells, such as regulatory T cells (Tregs), myeloid-derived suppressor cells (MDSCs), tumor-associated macrophages (TAMs), etc., are recruited to the tumor site to counteract excessive immune stimulation [31,32], but they also maintained tumor progression and turned the TME into an immunosuppressive microenvironment [30]. Multiple mechanisms of tumor immune evasion and metastasis have been implicated in the failure of certain clinical tumor immunotherapies [21]. Therefore, we urgently need to explore potential immune-escape related biomarkers, and improve the efficacy of immunotherapy.

In this study, we sought to explore genes closely associated with ccRCC among the 182 core cancer innate immune escape genes identified by Lawson et al. Firstly, we utilized the online database GSCA to screen mutated genes in ccRCC samples in the TCGA database, including amplification or deletion of CNV. We also used the ConsensusClusterPlus package in R software to cluster clinical samples from 4 cohorts, and then analyzed the differences in clinical prognosis, biological function, and immune microenvironment between IER A and IER B clusters. Next, we used the genes with the most significant differences between IER A and IER B clusters to perform secondary clustering on the clinical samples, and re-analyzed the prognosis and biological differences between geneCluster A and geneCluster B, so as to optimize the prognosis model. On this basis, we used lasso cox regression to calculate the immune escape score to explore the potential clinical significance. Meanwhile, we established a nomogram related to tumor immune escape and verified the stability of the nomogram in predicting the prognosis of ccRCC patients. Finally, we identified IFNAR1 as a key molecule associated with immune escape in ccRCC by external cohort validation. High expression of IFNAR1 was not only significantly associated with longer survival of patients, but also indicated a high infiltration of various immune cells, especially CD4^+^ T cells and CD8^+^ T cells. Previous studies also indicated that decreased expression of IFNAR1 in human colorectal carcinoma tissues resulted in the suppression of the cytotoxic T lymphocytes function [33], and the combination of Peg-IFNα with PD-1 blockade dramatically enhanced T-cell infiltration and improved the efficacy of PD-1 antibody on hepatocellular carcinoma [34]. Yang Lei et al. also found that IFNAR1 levels were significantly positively associated with T-cell infiltration and IFNAR1 may be a chemotherapy biomarker for predicting response [35].

Interferons (IFNs) act as regulatory cytokines against viral infection and can interfere with viral replication [36,37]. IFNs have important functions in coordinating adaptive and innate antitumor immune responses. Type I interferons (IFN-1), including interferon-α and interferon-β, have been shown to play a direct role in the control of cell growth and have potent immunomodulatory and antiangiogenic properties, which is a key medium in the immune response. Several reports have observed that the deletion or inactivation of STAT1 could destroy the IFN signal transduction [38], and STAT1 expression is associated with better prognosis, suggesting that the anti-tumor immunity of IFN may be achieved through the JAK/STAT pathway [39,40]. Stat1/Stat2/IRF9 complex needs to be formed and translocated to the nucleus in the signal transmission of IFN-α. Therefore, without Stat1, IFN type 1 responsive genes cannot be triggered. As one of the subunits constituting the type 1 interferon receptor, IFNAR1 has been confirmed to play a key role in the anti-tumor immunity of IFN-1. It has been reported that proline deletion in IFNAR1 could impair type I interferon signaling, thereby modulating the antitumor immune function of type I interferons [10]. Studies also have demonstrated that loss of IFNAR1 expression and JAK/STAT signaling may reduce NK cell-mediated antitumor immunity and thus promote breast cancer metastasis [41,42].

In conclusion, based on the overall expression pattern of immune escape-related genes, a prognostic model for predicting the overall survival of patients with renal clear cell carcinoma was established by using comprehensive methods, and the immune escape score was calculated. It was found that a higher immune escape score was significantly correlated with a shorter survival time. Meanwhile, through the validation of the external cohort and the correlation analysis of the immune microenvironment, we proved that IFNAR1 is the key gene regulating immune escape in ccRCC, and we also found that the function of IFNAR1 in promoting immune activation is achieved by facilitating the infiltration of CD4^+^ T cells and CD8^+^ T cells. Our research found a new key molecule that regulates the immune microenvironment of ccRCC, which could accurately predict the efficacy of ccRCC immunotherapy, thus providing a new idea for immunotherapy in ccRCC.

## Data Availability

DNA copy file and RNA-seq data, labeled TCGA-KIRC, were downloaded from (https://portal.gdc.cancer.gov/ (accessed on 25 March 2022)). EMBL database (https://www.ebi.ac.uk/arrayexpress/ (accessed on 15 March 2022)). The transcriptome data of RECA-EU were downloaded from ICGC (https://dcc.icgc.org/ (accessed on 15 March 2022)) database. Transcriptome data for ccRCC samples in the CPTAC database were downloaded from https://cptac-data-portal.georgetown.edu/study-summary/S050 (accessed on 6 March 2022).

## Supplementary Materials

The following supporting information can be downloaded at: https://www.mdpi.com/article/10.3390/cancers15010169/s1, Figure S1: Flow chart of this research; Figure S2: Supplementary figures for mIHC, Western Blot and IFNAR1 expression in CM-025 cohort; Table S1: core CTL-related genes; Table S2: quantification of CD4 CD8 IFNAR1.

## Abbreviations

AUC: area under curve; ccRCC: clear cell renal cell carcinoma; CNV: copy number variations; DEG: differentially expressed genes; GSVA: gene set variation analysis; OS: overall survival; ROC: receiver operating characteristic; ssGSEA: single sample gene set enrichment analysis; TCGA: the cancer genome atlas; TME: tumor microenvironment.

## References

[1] Xie Y., Xie F., Zhang L., Zhou X., Huang J., Wang F., Jin J., Zhang L., Zeng L., Zhou F. (2021). Targeted Anti-Tumor Immunotherapy Using Tumor Infiltrating Cells. Adv. Sci..

[2] Lawson K.A., Sousa C.M., Zhang X., Kim E., Akthar R., Caumanns J.J., Yao Y., Mikolajewicz N., Ross C., Brown K.R. (2020). Functional genomic landscape of cancer-intrinsic evasion of killing by T cells. Nature.

[3] Deleuze A., Saout J., Dugay F., Peyronnet B., Mathieu R., Verhoest G., Bensalah K., Crouzet L., Laguerre B., Belaud-Rotureau M.-A. (2020). Immunotherapy in Renal Cell Carcinoma: The Future Is Now. Int. J. Mol. Sci..

[4] Ljungberg B., Campbell S.C., Cho H.Y., Jacqmin D., Lee J.E., Weikert S., Kiemeney L.A. (2011). The epidemiology of renal cell carcinoma. Eur. Urol..

[5] Wagstaff J., Jones R., Hawkins R., Porfiri E., Pickering L., Bahl A., Brown J., Buchan S. (2016). Treatment patterns and clinical outcomes in patients with renal cell carcinoma in the UK: Insights from the RECCORD registry. Ann. Oncol..

[6] Abe H., Kamai T. (2013). Recent advances in the treatment of metastatic renal cell carcinoma. Int. J. Urol..

[7] Mathew L.K., Lee S.S., Skuli N., Rao S., Keith B., Nathanson K.L., Lal P., Simon M.C. (2014). Restricted expression of *miR-30c-2-3p* and *miR-30a-3p* in clear cell renal cell carcinomas enhances HIF2α activity. Cancer Discov..

[8] Eng L., Ding D., Chen Y., Dai H., Liu G., Qiao Z., An R. (2014). Anti-tumor effect of ribavirin in combination with interferon-α on renal cell carcinoma cell lines in vitro. Cancer Cell Int..

[9] Parker B.S., Rautela J., Hertzog P.J. (2016). Antitumour actions of interferons: Implications for cancer therapy. Nat. Rev. Cancer.

[10] Zhang G., Deweerd N.A., Stifter S.A., Liu L., Zhou B., Wang W., Zhou Y., Ying B., Hu X., Matthews A.Y. (2018). A proline deletion in IFNAR1 impairs IFN-signaling and underlies increased resistance to tuberculosis in humans. Nat. Commun..

[11] Leek J.T., Johnson W.E., Parker H.S., Jaffe A.E., Storey J.D. (2012). The sva package for removing batch effects and other unwanted variation in high-throughput experiments. Bioinformatics.

[12] Liu C.-J., Hu F.-F., Xia M.-X., Han L., Zhang Q., Guo A.-Y. (2018). GSCALite: A web server for gene set cancer analysis. Bioinformatics.

[13] Wilkerson M.D., Hayes D.N. (2010). ConsensusClusterPlus: A class discovery tool with confidence assessments and item tracking. Bioinformatics.

[14] Hänzelmann S., Castelo R., Guinney J. (2013). GSVA: Gene set variation analysis for microarray and RNA-Seq data. BMC Bioinform..

[15] Ritchie M.E., Belinda P., Wu D., Hu Y., Law C.W., Shi W., Smyth G.K. (2015). limma powers differential expression analyses for RNA-sequencing and microarray studies. Nucleic Acids Res..

[16] Gu Z., Eils R., Schlesner M. (2016). Complex heatmaps reveal patterns and correlations in multidimensional genomic data. Bioinformatics.

[17] Yu G., Wang L.-G., Han Y., He Q.-Y. (2012). clusterProfiler: An R package for comparing biological themes among gene clusters. OMICS J. Integr. Biol..

[18] Braun D.A., Hou Y., Bakouny Z., Ficial M., Angelo M.S., Forman J., Ross-Macdonald P., Berger A.C., Jegede O.A., Elagina L. (2020). Interplay of somatic alterations and immune infiltration modulates response to PD-1 blockade in advanced clear cell renal cell carcinoma. Nat. Med..

[19] Ru B., Wong C.N., Tong Y., Zhong J.Y., Zhong S.S.W., Wu W.C., Chu K.C., Wong C.Y., Lau C.Y., Chen I. (2019). TISIDB: An integrated repository portal for tumor–immune system interactions. Bioinformatics.

[20] Li T., Fu J., Zeng Z., Cohen D., Li J., Chen Q., Li B., Liu X.S. (2020). TIMER2.0 for analysis of tumor-infiltrating immune cells. Nucleic Acids Res..

[21] Liu Y., Cao X. (2016). Immunosuppressive cells in tumor immune escape and metastasis. J. Mol. Med..

[22] Kitamura T., Qian B.-Z., Pollard J.W. (2015). Immune cell promotion of metastasis. Nat. Rev. Immunol..

[23] Perdiguero E.G., Geissmann F. (2014). Identifying the infiltrators. Science.

[24] Kim R., Emi M., Tanabe K. (2007). Cancer immunoediting from immune surveillance to immune escape. Immunology.

[25] Schreiber R.D., Old L.J., Smyth M.J. (2011). Cancer immunoediting: Integrating immunity’s roles in cancer suppression and promotion. Science.

[26] Vinay D.S., Ryan E.P., Pawelec G., Talib W.H., Stagg J., Elkord E., Lichtor T., Decker W.K., Whelan R.L., Kumara H.M.C.S. (2015). Immune evasion in cancer: Mechanistic basis and therapeutic strategies. Semin. Cancer Biol..

[27] Jiang X., Wang J., Deng X., Xiong F., Ge J., Xiang B., Wu X., Ma J., Zhou M., Li X. (2019). Role of the tumor microenvironment in PD-L1/PD-1-mediated tumor immune escape. Mol. Cancer.

[28] Jago C.B., Yates J., Câmara N.O.S., Lechler R.I., Lombardi G. (2004). Differential expression of CTLA-4 among T cell subsets. Clin. Exp. Immunol..

[29] Blackburn S.D., Shin H., Haining W.N., Zou T., Workman C.J., Polley A., Betts M.R., Freeman G.J., Vignali D.A., Wherry E.J. (2008). Coregulation of CD8+ T cell exhaustion by multiple inhibitory receptors during chronic viral infection. Nat. Immunol..

[30] Huber V., Camisaschi C., Berzi A., Ferro S., Lugini L., Triulzi T., Tuccitto A., Tagliabue E., Castelli C., Rivoltini L. (2017). Cancer acidity: An ultimate frontier of tumor immune escape and a novel target of immunomodulation. Semin. Cancer Biol..

[31] Oleinika K., Nibbs R.J., Graham G.J., Fraser A.R. (2013). Suppression, subversion and escape: The role of regulatory T cells in cancer progression. Clin. Exp. Immunol..

[32] Gabrilovich D.I., Nagaraj S. (2009). Myeloid-derived suppressor cells as regulators of the immune system. Nat. Rev. Immunol..

[33] Lu C., Klement J.D., Ibrahim M.L., Xiao W., Redd P.S., Nayak-Kapoor A., Zhou G., Liu K. (2019). Type I interferon suppresses tumor growth through activating the STAT3-granzyme B pathway in tumor-infiltrating cytotoxic T lymphocytes. J. Immunother. Cancer.

[34] Zhu Y., Chen M., Da Xu D., Li T.-E., Zhang Z., Li J.-H., Wang X.-Y., Yang X., Lu L., Jia H.-L. (2022). The combination of PD-1 blockade with interferon-α has a synergistic effect on hepatocellular carcinoma. Cell. Mol. Immunol..

[35] Yang L., Zhang X., Huang X., Dong X., Jing S., Zhang Y., Zhao B., Wang Z., Qu H. (2022). Correlation between IFNAR1 expression in peripheral blood T lymphocytes and inflammatory cytokines, tumor-infiltrating lymphocytes, and chemosensitivity in patients with colorectal cancer. Cytokine.

[36] Isaacs A., Lindenmann J. (1957). Virus interference: I. The interferon. Proc. R. Soc. Lond. Ser. B-Biol. Sci..

[37] Von Locquenghien M., Rozalén C., Celià-Terrassa T. (2021). Interferons in cancer immunoediting: Sculpting metastasis and immunotherapy response. J. Clin. Investig..

[38] Adámková L., Součkova K., Kovarík J. (2007). Transcription protein STAT1: Biology and relation to cancer. Folia Biol..

[39] Widschwendter A., Tonko-Geymayer S., Welte T., Daxenbichler G., Marth C., Doppler W. (2002). Prognostic significance of signal transducer and activator of transcription 1 activation in breast cancer. Clin. Cancer Res..

[40] Simpson J.A.D., Al-Attar A., Watson N.F.S., Scholefield J.H., Ilyas M., Durrant L.G. (2010). Intratumoral T cell infiltration, MHC class I and STAT1 as biomarkers of good prognosis in colorectal cancer. Gut.

[41] Bottos A., Gotthardt D., Gill J.W., Gattelli A., Frei A., Tzankov A., Sexl V., Wodnar-Filipowicz A., Hynes N.E. (2016). Decreased NK-cell tumour immunosurveillance consequent to JAK inhibition enhances metastasis in breast cancer models. Nat. Commun..

[42] Rautela J., Baschuk N., Slaney C.Y., Jayatilleke K.M., Xiao K., Bidwell B.N., Lucas E.C., Hawkins E.D., Lock P., Wong C.S. (2015). Loss of Host Type-I IFN Signaling Accelerates Metastasis and Impairs NK-cell Antitumor Function in Multiple Models of Breast Cancer. Cancer Immunol. Res..

