# Supplementary material for: Construction of an Immune Escape-Related Signature in Clear Cell Renal Cell Carcinoma and Identification of the Relationship between IFNAR1 and Immune Infiltration by Multiple Immunohistochemistry
Source: Cancers (Basel). 2022 Dec 28;15(1):169. doi: 10.3390/cancers15010169 (PMC9818644; doi:10.3390/cancers15010169)
Supplement: Supplementary file 1 [file cancers-15-00169-s001.zip › Uncropped Western blot figures.pdf]

Supplementary Materials

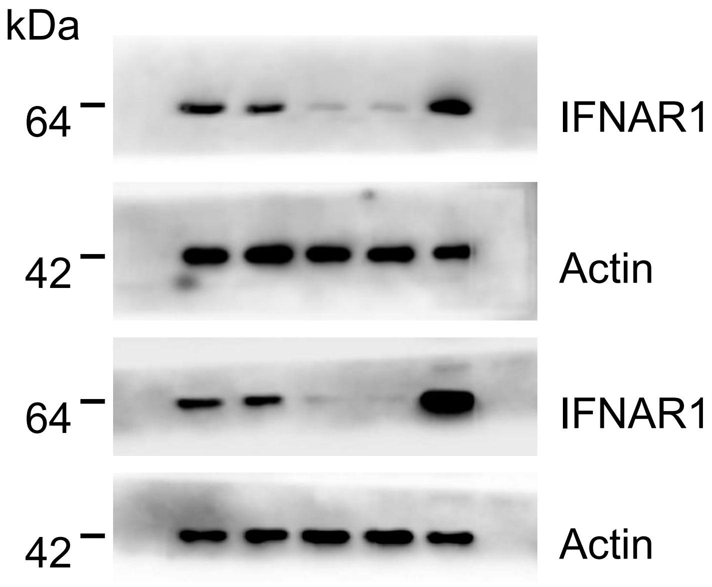

The uncropped Western blot figures related to Figure 8A.

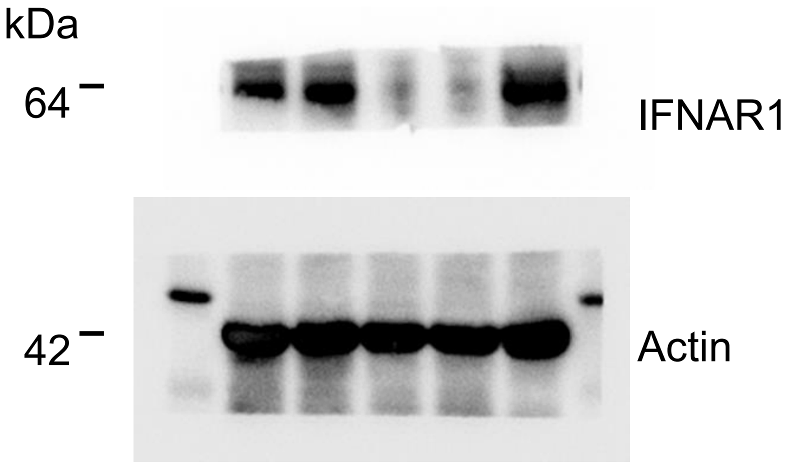

The uncropped Western blot figures related to Supplementary figure2C.
